# Genome Sequence of Bacillus anthracis Isolated from an Anthrax Burial Site in Pollino National Park, Basilicata Region (Southern Italy)

**DOI:** 10.1128/genomeA.00141-15

**Published:** 2015-03-19

**Authors:** Antonio Fasanella, Peter Braun, Gregor Grass, Matthias Hanczaruk, Angela Aceti, Luigina Serrecchia, Giuseppe Leonzio, Francesco Tolve, Enrico Georgi, Markus Antwerpen

**Affiliations:** a Istituto Zooprofilattico Sperimentale of Puglia and Basilicata, Anthrax Reference Institute of Italy, Foggia, Italy; b Bundeswehr Institute of Microbiology, Munich, Germany

## Abstract

A Bacillus anthracis strain was isolated from a burial-site in Pollino National Park where a bovine died of anthrax and was buried in 2004. We report the first genome sequence of B. anthracis isolated in the Basilicata region (southern Italy), which is the highest risk area of anthrax infection in Italy.

## GENOME ANNOUNCEMENT

Animal anthrax is endemic in Italy and is characterized by sporadic outbreaks that tend to occur during summer. With the exception of rare outbreaks, the disease is predominantly concentrated in the southern and island regions. Over the last 20 years, 121 outbreaks of animal anthrax were recorded. The most affected region is Basilicata with 62 outbreaks (1). At the end of the summer of 2004 the area of Pollino National Park of Basilicata was hit by a severe epidemic that afflicted both livestock and wildlife. A total of 126 animals, mostly cattle but also horses, sheep, goats, and red deer died within 40 days (2, 3). There were two suspect cutaneous human cases. Tabanid flies as vectors for the spread of the disease were hypothesized (4).

In the spring of 2014 we revisited a known bovine burial site of the outbreak at Pollino National Park and isolated B. anthracis from a soil sample drawn from near the surface (at 5 cm depth), approximately 100 cm above a buried bovine carcass. In accordance with its virulent phenotype, initial characterization of the isolate named Pollino revealed the presence of both virulence plasmids (pXO1 and pXO2). Genotyping clustered the strain within a subgroup (A.Br.011/009) of the wide-spread trans-Eurasian (TEA) clade (5) of B. anthracis, which is typical for large areas of Europe and Asia and is predominant for Italy (previously determined as Cluster A1) (2, 3).

The genome sequence of the strain Pollino is the first genome sequence reported from the Basilicata region and can be expected to serve as a reference for follow-up studies on the spread of closely related B. anthracis genotypes in Italy and for making inferences about the genetic relationships of presently cultured or future outbreak isolates from within Pollino National Park or from surrounding areas of southern Italy.

Whole-genome shotgun (WGS) sequencing of B. anthracis Pollino was performed by Ion Torrent sequencing technology (Ion Torrent Systems, Inc., USA). For the WGS library, 2,274,452 reads with a total of 545 Mb were generated. Using bowtie2-software (6), 98.81% of the reads were mapped to Ames Ancestor chromosome, plasmid pXO1 and pXO2 (NC_007530.2, NC_007322.2, AE017335.3), respectively. The G+C content was calculated using an in-house Python script. Unmapped reads were *de novo* assembled using the velvet algorithm (7) and resulting contigs were analyzed. No significant hits were found after blasting these remaining reads against the non-redundant (nr) nucleotide database of NCBI.

The total length of the genome shotgun sequence of B. anthracis Pollino was 5,227,390 bp with a coverage of 111-fold, and the mean G+C content was 33.7%. The plasmids pXO1 and pXO2 yielded sizes of 181,650 and 94,7802 bp and with coverages of 272- and 146-fold, respectively. The transfer utility of Geneious 7.01 software (Biomatters) was used for annotation.

The genome encodes 5,295 putative coding sequences (CDSs, 5,019, with 178 and 98 on the chromosomes pXO1 and pXO2, respectively). In the final genome sequence eleven copies of the 16S rRNAs, 5S rRNAs, and 23S rRNAs were identified, as well as 94 tRNAs loci.

### Nucleotide sequence accession numbers.

This genome project has been deposited at DDBJ/EMBL/GenBank under the accession numbers CP010813, CP010814, and CP010815. The versions described in this paper are the ﬁrst versions.
